# ACE2: The Key Molecule for Understanding the Pathophysiology of Severe and Critical Conditions of COVID-19: Demon or Angel?

**DOI:** 10.3390/v12050491

**Published:** 2020-04-28

**Authors:** Li Xiao, Hiroshi Sakagami, Nobuhiko Miwa

**Affiliations:** 1 Department of Pharmacology, School of Life Dentistry at Tokyo, The Nippon Dental University, Tokyo 102-0071, Japan; 2 Meikai University Research Institute of Odontology (M-RIO), Saitama 3500283, Japan; 3 Faculty of Life Sciences, Prefectural University of Hiroshima, Hiroshima 7270023, Japan

**Keywords:** COVID-19, ACE2, RAAS, Ang-(1-7), SARS-CoV-2, ADAM17, TMPRSS2, B^0^AT1

## Abstract

Recently, the SARS-CoV-2 induced disease COVID-19 has spread all over the world. Nearly 20% of the patients have severe or critical conditions. SARS-CoV-2 exploits ACE2 for host cell entry. ACE2 plays an essential role in the renin–angiotensin–aldosterone system (RAAS), which regulates blood pressure and fluid balance. ACE2 also protects organs from inflammatory injuries and regulates intestinal functions. ACE2 can be shed by two proteases, ADAM17 and TMPRSS2. TMPRSS2-cleaved ACE2 allows SARS-CoV-2 cell entry, whereas ADAM17-cleaved ACE2 offers protection to organs. SARS-CoV-2 infection-caused ACE2 dysfunction worsens COVID-19 and could initiate multi-organ failure. Here, we will explain the role of ACE2 in the pathogenesis of severe and critical conditions of COVID-19 and discuss auspicious strategies for controlling the disease.

## 1. Introduction

Since December 2019, the new coronavirus (severe acute respiratory syndrome coronavirus 2, SARS-CoV-2) induced disease, COVID-19, has spread rapidly all over the planet. As shown in COVID-19 Dashboard by the Center for Systems Science and Engineering (CSSE) at Johns Hopkins University (JHU), up to date 2,703,615 people have been infected and 190,490 deaths have been confirmed worldwide [1].WHO officially declared COVID-19 a pandemic on March 12th, 2020. According to a report based on 72,314 cases (test confirmed cases: 44,672 (62%) from the Chinese Center for Disease Control and Prevention, 81% of COVID-19 patients have cold-like symptoms and mild pneumonia, 14% have severe respiratory inflammation, and 5% have critical conditions including respiratory failure, septic shock, and/or multiple organ dysfunction or failure. The mortality is 2.3% (49.0% in critical cases) [2]. Since the rates of severe and critical cases are much higher than seasonal influenza, it is essential to understand the pathophysiology and then devise strategies to fight the disease.

SARS-CoV-2 is a positive-sense single-stranded RNA virus with a crown-like appearance of spike proteins (S proteins) that project from their envelope. Similar to SARS (severe acute respiratory syndrome, 2002–2003) coronavirus (SARS-CoV) [3], SARS-CoV-2 primarily uses the S protein to invade host cells through ACE2, an enzyme which is known to be important in the renin–angiotensin–aldosterone system (RAAS) [4,5].

## 2. Profile of ACE2

Human angiotensin-converting enzyme-related carboxypeptidase ACE2 is encoded by the ACE2 gene which maps to chromosome Xp22 [6]. ACE2 is a type I transmembrane protein, comprised of an extracellular heavily N-glycosylated N-terminal domain containing the carboxypeptidase site and a short intracellular C-terminal cytoplasmic tail [7]. The N-terminal peptidase domain is also the SARS-CoV binding site [8]. There are two forms of ACE2 protein: cellular (membrane-bound) form and circulating (soluble) form. Cellular ACE2 protein is the full-length protein which is expressed abundantly in pneumocytes and enterocytes of the small intestine [9]. ACE2 is also expressed in vascular endothelial cells of the heart, the kidneys, and other organs, such as the brain. However, ACE2 is absent in the spleen, thymus, lymph nodes, bone marrow, and cells of the immune system (including B and T lymphocytes, and macrophages) [10,11]. 

**Figure 1:**
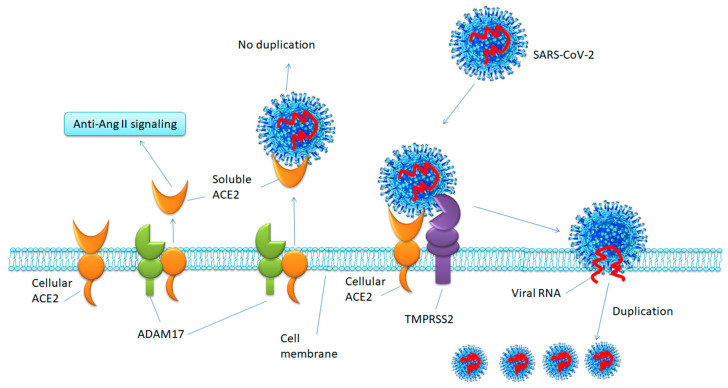
Hypothesis of ACE2 shedding and SARS-CoV-2 entry.

Circulating ACE2 (with the N-terminal peptidase domain) is cleaved from the full-length ACE2 on the cell membrane by the metalloprotease ADAM17 and then released into the extracellular environment [7]. The type II transmembrane serine protease, TMPRSS2 was found to compete with ADAM17 for ACE2 shedding but cleaves ACE2 differently. Both ADAM17 and TMPRSS2 remove a short C-terminal fragment from ACE2. Arginine and lysine residues within amino acids 652 to 659 are critical for ADAM17 shedding, whereas arginine and lysine residues within amino acids 697 to 716 are essential for TMPRSS2 shedding. Only cleavage by TMPRSS2 results in augmented SARS-CoV cell entry [12,13,14,15]. There are two ways for SARS-CoV to enter the target cell: endocytosis, and fusion of the viral membrane with a membrane of the target cell, which is 100 times more efficient than endocytosis for viral replication [16]. With the help of TMPRSS2, ADAM17-regulated ectodomain shedding of ACE2 could induce SARS-CoV cell entry through endocytosis [7,12]; however, ADAM17 activity is not required for SARS-CoV cell entry through fusion [12]. As the N-terminal domain is the coronavirus binding site, circulating ACE2 also binds to the virus. Iwata-Yoshikawa et al. infected both wild type (WT) and TMPRSS2 knockout (KO) mice with SARS-CoV. Their results showed that compared to WT mice, SARS-CoV-infected TMPRSS2 KO mice expressed lower viral replication in the lungs with much lighter alveolar damage and less cytokines release [17]. Recently, two research groups demonstrated that intracellular entry of SARS-CoV-2 also depends on TMPRSS2-shed ACE2 [4,5]. TMPRSS2 is highly expressed in large populations of epithelial cells (prostate > colon > small intestine > pancreas > kidney > lung > liver) and is upregulated by androgen [18,19]. In human respiratory and intestinal cells, less than 10% of the cells (including lung type II pneumocytes, ileal absorptive enterocytes, and nasal goblet secretory cells) co-express ACE2 and TMPRSS2 [20]. This evidence indicates that for successful SARS-CoV-2 infection, cellular ACE2 and TMPRSS2 are necessary (Figure 1), and male sex hormones may contribute to the infectibility of the host (a recent clinical study reported that among 191 COVID-19 inpatients, 62% were male; among non-survivors 70% were male [21]). In contrast, the major shedding enzyme, ADAM17-shedded ACE2 (circulating ACE2) is believed to protect lungs from the viral infection [12]. Evidence has shown that the expression of TMPRSS2 inhibits ADAM17-shedding of ACE2 [12]. However, it is unclear how TMPRSS2 transcends ADAM17 to cleave ACE2 during SARS-CoV or SARS-CoV-2 infection.

Cellular ACE2, ADAM17, and TMPRSS2 are all expressed on the cell membrane. After being shed by ADAM17, soluble ACE2 is released from its full-length form to counteract the effects of Ang II signaling. Alternatively, cellular ACE2 is also shed by TMPRSS2, which results in SARS-CoV-2-cell membrane fusion. SARS-CoV-2 RNA is then released into the cytoplasm and viral replication is efficiently processed. Because soluble ACE2 contains the virus binding site, it can also bind to the virus. However, without an intracellular environment, the virus cannot be duplicated.

## 3. Functions of ACE2 

**Figure 2:**
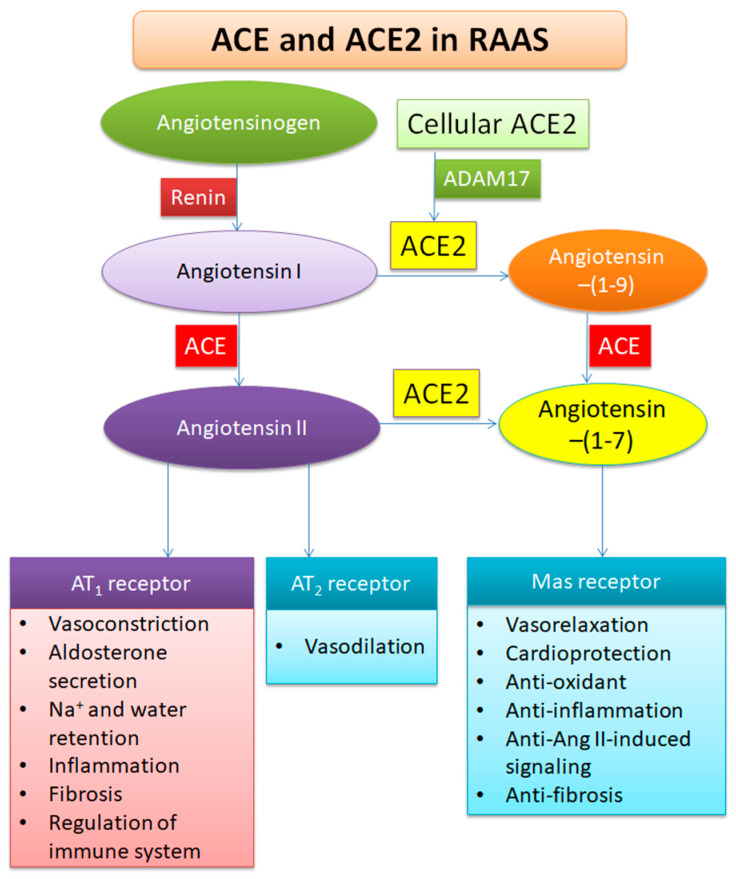
Role of ACE2 in the renin–angiotensin–aldosterone system (RAAS).

ACE2 is a close homologue of human ACE [22,23]. As is well-known, ACE cleaves angiotensin I (Ang I) to Ang II. Ang II binds to Ang II receptor 1 (AT_1_) and then mediates numerous systemic and local effects (such as promoting vasoconstriction, fibrosis, and salt retention) in the cardiovascular system (Figure 2). In the RAAS, ACE2 exerts the opposite role of ACE. ACE2 catalyzes the conversion of Ang I to Ang-(1-9) and Ang II to Ang-(1-7). The converting efficiency of ACE2 on substrate Ang II is 400-fold higher than that on Ang I [24]. Ang-(1-7) binds to the G protein-coupled receptor Mas to mediate various effects including vasorelaxation, cardioprotection, anti-oxidative action [25], anti-inflammation [26], and the inhibition of Ang II-induced signaling [27,28]. The ACE2-Ang-(1-7) axis is considered an important therapeutic target in cardiovascular disorders [29]. A large cohort study showed that circulating ACE2 was only detectable in the serum of 40 among 534 test subjects, and its concentration was approximately 100-fold lower than that of circulating ACE [30]. More evidence showed that circulating ACE2 is increased in patients with type 1 or type 2 diabetes, hypertension, heart failure, and chronic kidney diseases [31,32,33]. The reason for high levels of ACE2 in these patients is that increased ACE2 is a defensive response to counteract the adverse effect of Ang II. Since Ang II-AT_1_ receptor signaling also promotes autoimmune response, ACE2 may control immune functions through the Ang-(1-7)-Mas axis [34,35].

Angiotensinogen is produced in the liver and released into the blood stream. The renal juxtaglomerular apparatus-secreted renin cleaves angiotensinogen to Ang I. Ang I is further converted to Ang II by ACE which is mostly produced in the lungs. Ang II binds to both AT_1_ and AT_2_ receptors to regulate the blood pressure and inflammation. Most actions of Ang II occur via the AT_1_ receptor. Meanwhile, cellular ACE2 is cleaved by ADAM17. After the active form of ACE2 being released into the extracellular environment, ACE2 converts Ang I to Ang-(1-9) and Ang II to Ang-(1-7). ACE also converts Ang-(1-9) to Ang-(1-7). Ang-(1-7) binds to Mas receptor to mediate the opposite effects of Ang II.

Genetic ACE2 deficiency is associated with upregulation of inflammatory mediators, elevated inflammatory responsiveness to proinflammatory stimuli, and enhanced Ang II-induced cardiac and aortic remodeling [36,37]. The anti-inflammatory effects of ACE2 are exerted mostly through the ACE2-Ang-(1-7) axis against Ang II-AT_1_ activities [38].

ACE2 also has a RAAS-independent function. In the intestine, cellular ACE2 (not circulating ACE2) regulates the absorption of amino acids and the intestinal bacterial balance to reduce intestinal inflammation [39]. Cellular ACE2 is required for expression of the neutral amino acid transporter B^0^AT1 in intestinal epithelial cells [40]. Without ACE2-B^0^AT1 complex, serum levels of the neutral amino acids valine (Val), threonine (Thr), and tyrosine (Tyr), and the essential amino acid tryptophan (Trp) were markedly reduced and resulted in severe intestinal inflammation and microbial imbalance in ACE2 knockout mice. Administration with Trp or nicotinamide (the metabolic product of Trp) could increase the expression of antimicrobial peptide α-defensins and reverse the above-mentioned microbial imbalance and inflammation in the gut. Structural modelling suggested that the ACE2-B^0^AT1 complex could bind to the S-protein of SARS-CoV-2 simultaneously [41]. The intestinal cellular ACE2 might be another viral entry point for SARS-CoV-2 as well as TMPRSS2-sheded ACE2 in the lungs.

## 4. Anatomic and Pathological Findings of Severe and Critical COVID-19: What is the Role of ACE2?

**Figure 3:**
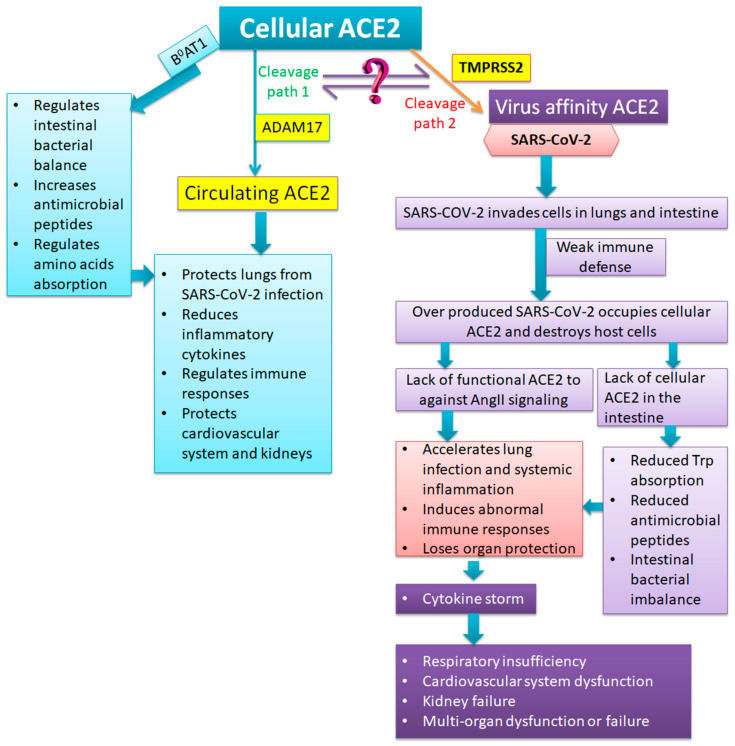
Hypothesis of ACE2 and the pathogenesis of severe and critical COVID-19.

COVID-19 is a lower respiratory tract disease. The first anatomic pathology report of a COVID-19 death indicated that the most seriously injured organs were the lungs. Exudative lesions and fibrosis were observed in the lungs. Phlegm and exudates filled up the lower respiratory tract and pulmonary alveolus. Compared to SARS, the exudative lesions were far worse but the fibrosis was much lighter. Notably, segmental dilatation and stenosis of the small intestine were observed in the cadaver suggesting that the small intestine was also severely injured by SARS-CoV-2 infection. Lesions on other organs were not obvious [42]. The pathological findings of another COVID-19 victim showed that the bilateral diffuse alveolar damage with cellular fibromyxoid exudates, desquamation of pneumocytes, and hyaline membrane formation occurred in the lungs [43]. This evidence suggests that SARS-CoV-2 invades the human body mostly through the respiration system, and possibly also through the intestine and other tissues. In 2004, Ding et al. detected organ distribution of SARS-CoV by immunohistochemistry and in situ hybridization. SARS-CoV was mainly found in the respiratory system, stomach, small intestine, kidneys, and sweat glands [44]. The organ distribution of SARS-CoV-2 is probably similar to SARS-CoV. Based on the evidence described above, we hypothesize that SARS-CoV-2 invades the lungs and intestine through TMPRSS2-cleaved ACE2. If the immune system is not able to defeat the infection, SARS-CoV-2 will be massively replicated, occupy cellular ACE2, and destroy the host’s cells. As a consequence, the Ang II-AT_1_ signaling cannot be inactive. Together, the intestinal function is ruined and the inflammation exacerbated. As a result, a cytokine storm occurs and eventually the respiration system, cardiovascular system and other organs lose function (Figure 3). Clinical data showed that among COVID-19 inpatients, about 30% have underlying diseases. These patients have an increased risk of death. Hypertension was the most common, followed by diabetes and coronary heart disease [21,45]. Since the Ang II-AT_1_ axis is hyperactive in these diseases already [46,47], SARS-CoV-2 further decreases the production of functional ACE2. Therefore, in patients with those underlying diseases it is much easier to develop severe and critical conditions.

Cellular ACE2 can be shed by both ADAM17 and TMPRSS2. The ADAM17-cleavage is a normal path which results in the production of circulating ACE2. Circulating ACE2 can prevent severe pathological conditions and protect organs during SARS-CoV-2 infection. In contrast, TMPRSS2-shed ACE2 allows the SARS-CoV-2 to invade cells in the lungs and intestine. TMPRSS2-cleavage path might inhibit ADAM17-cleavage path. If the immune system is not able to defeat the virus, SARS-CoV-2 will be massively reproduced, occupy cellular ACE2, and destroy the host’s cells in the lungs and intestine. As SARS-CoV-2 reduces ACE2 expression, there is not enough ADAM17-shed circulating ACE2 against the Ang II signaling-induced inflammatory injuries, and inflammation is accelerated until the immune system is overwhelmed. Meanwhile, cellular ACE2/B^0^AT1 in the intestine is ruined by the virus. Essential amino acids cannot be absorbed, the antimicrobial peptides are reduced, and the ecology of the gut microbiome is damaged. These intestinal changes will precipitate inflammation. As a result, a cytokine storm occurs and eventually induces multiple organ dysfunction or failure.

## 5. Strategies for Preventing and Reversing Severe and Critical Conditions of COVID-19

As addressed above, the strategies for preventing and reversing severe and critical COVID-19 should include:

### 5.1. Inhibiting TMPRSS2 to Block SARS-CoV-2 Cell Entry 

Since TMPRSS2 plays a very important role in SARS-CoV-2 cell entry and ACE2 dysfunction, blocking the activity of TMPRSS2 should be the primary strategy for preventing severe and critical conditions of COVID-19. Hoffmann et al. discovered that a serine protease inhibitor, camostat mesylate, partially blocked TMPRSS2-ACE2-mediated SARS-CoV-2 entry [3]. Similarly, nafamostat mesylate inhibited the TMPRSS2-ACE2-mediated SARS-CoV-2 envelope-plasma membrane fusion, and then showed 10 times higher efficiency than camostat mesylate in preventing SARS-CoV-2 cell entry [48]. Both camostat mesylate and nagamostat mesylate are clinically approved medicines with verified safety, and thus can be applied for COVID-19 treatment in clinical practices immediately. Nafamostat mesylate also has been used as a short-acting anticoagulant due to its ability to prevent the proteolysis of fibrinogen into fibrin. Clinical reports of COVID-19 showed that increased D-dimer (a fibrin degradation product) levels in severe and critical patients were often observed [21,49,50]. High levels of D-dimer (>1 μg/mL) are related to higher risk of mortality [19]. Thus, nafamostat mesylate not only can block the viral entry, but also prevent thrombosis and disseminated intravascular coagulation (DIC) in COVID-19 patients. A clinical trial of COVID-19 with nafamostat mesylate treatment in Japan started in March 2020.

### 5.2. Increasing ACE2 or Ang-(1-7)

Because SARS-CoV-2 exploits ACE2 for entry, some scientists are concerned that the expression of ACE2 might provide possible advantageous routes for virus entry. They suggest that patients with hypertension, diabetes, and cardiovascular diseases should reduce the use of ACE inhibitors and Ang II-AT1 blockers because these medicines can increase the expression of ACE2 [51]. Ibuprofen and thiazolidinediones were also suspected of increasing the risk of COVID-19 for the same reason [52]. However, there is no evidence that those medicines can facilitate SARS-CoV or SARS-CoV-2 infection. On the contrary, SARS-CoV infection could reduce ACE2 expression and worsen acute lung failure, which could be attenuated by blocking the RAAS pathway [53]. ACE2 expression was also shown to protect against severe acute lung failure in a mouse model [54]. In addition, cell tropism of SARS-CoV12 and SARS-CoV-2 did not firmly associate with ACE2 expression. ACE2 is expressed on both type I and type II pneumocytes, whereas SARS-CoV and SARS-CoV-2 only make use of type II pneumocytes because the infection requires co-expression of ACE2 and TMPRSS2 as we described before [20,55,56]. Therefore, increasing ACE2 (especially circulating ACE2) or Ang-(1-7) is a potential approach to reduce SARS-CoV-2-induced severe damage and to protect organs. Recently, an international research team showed that clinical-grade human soluble ACE2 could combine with SARS-CoV-2 and decrease its infection rate to 10005000– times in engineered human blood vessel organoids and human kidney organoids [57].

### 5.3. Supplementation of Essential Amino Acids

Evidence showed that administration with Trp or nicotinamide could reverse severe intestinal inflammation in ACE2 knockout mice [39]. As a nutrient enhancer, Trp and its metabolites play crucial roles in gut microbiota maintenance, microbial metabolism, the host’s immune system, the host-microbiome interface, and host immune system-intestinal microbiota interactions. [58]. Thus, supplementation with Trp or nicotinamide may regulate the gut microbiome and increase antimicrobial peptides to convert SARS-CoV-2-induced lesions in the intestine and further improve systemic conditions. 

In summary, ACE2 plays essential roles in SARS-CoV-2 cell entry and makes an impact on the progress and prognosis of severe and critical conditions of COVID-19. Regulating ACE2-related enzymes and amino acid intake would be desirable for disease control. However, more experimental and clinical studies are required. Moreover, pathogenesis of severe and critical conditions of COVID-19 is very complicated. Other molecules, such as IL-6, also play important roles in the disease. Therefore, therapeutic strategies should be flexibly changed according to the patient’s clinical inspection results.
